# Biocompatibility of Bacterial Cellulose Based Biomaterials

**DOI:** 10.3390/jfb3040864

**Published:** 2012-12-05

**Authors:** Fernando G. Torres, Solene Commeaux, Omar P. Troncoso

**Affiliations:** 1 Polymers and Composites Group, Catholic University of Peru (PUCP), Lima 32, Peru; Email: troncoso.op@pucp.pe; 2 Polytech Montpellier, University of Montpellier, Montpellier 34095, France; Email: solene.commeaux@polytech.univ-montp2.fr

**Keywords:** bacterial cellulose, biocompatibility, biomedical application, biomaterials

## Abstract

Some bacteria can synthesize cellulose when they are cultivated under adequate conditions. These bacteria produce a mat of cellulose on the top of the culture medium, which is formed by a three-dimensional coherent network of pure cellulose nanofibers. Bacterial cellulose (BC) has been widely used in different fields, such as the paper industry, electronics and tissue engineering due to its remarkable mechanical properties, conformability and porosity. Nanocomposites based on BC have received much attention, because of the possibility of combining the good properties of BC with other materials for specific applications. BC nanocomposites can be processed either in a static or an agitated medium. The fabrication of BC nanocomposites in static media can be carried out while keeping the original mat structure obtained after the synthesis to form the final nanocomposite or by altering the culture media with other components. The present article reviews the issue of biocompatibility of BC and BC nanocomposites. Biomedical aspects, such as surface modification for improving cell adhesion, *in vitro* and *in vivo* studies are given along with details concerning the physics of network formation and the changes that occur in the cellulose networks due to the presence of a second phase. The relevance of biocompatibility studies for the development of BC-based materials in bone, skin and cardiovascular tissue engineering is also discussed.

## 1. Introduction

Cellulose is the most abundant biomass material on earth [1]. It forms the basic structural matrix for plant cell walls. It is mainly used in the textile and paper industry, and it is obtained from vegetal products. 

Bacterial cellulose (BC) is a type of cellulose synthesized by some bacteria. In stationary culture conditions, these bacteria produce a thick gel or pellicle of cellulose on the surface of the liquid medium. BC differs from plant cellulose in its higher purity, crystallinity, degree of polymerization and tensile strength [2,3,4,5]. BC has Iα and Iβ crystalline forms, unlike the cellulose of plants that present mainly the Iβ structure [6].

Traditionally, BC is used as raw material for a Filipino dessert called “nata-de-coco” [7], and it is also present in the mat used for the preparation of a beverage known as “kombucha tea” [2,8]. Biocompatibility of BC-based products have made them suitable for several biomedical applications, including membranes for wound dressings [9], scaffolds for tissue engineering [10,11,12,13], substrates for cell seeding [14,15], structures for biomineralization of hydroxyapatite [16], *etc.*

In this paper, we review the use of BC and BC-based structures as biomaterials and discuss issues related to their biocompatibility. First, we present the main characteristics of BC structures, including their morphology, their mechanical properties, as well as the processing routes used for their production. Then, we discuss the properties of BC as a biomaterial, including its rheological properties in the humid state, biocompatibility and biodegradability. The following section deals with the techniques used for improving the biocompatibility of BC. Some examples of surface modification and preparation of BC composites are given. Finally, we give several examples of BC-based structures for biomedical and clinical applications, with details concerning the biocompatibility achieved in each case.

## 2. Bacterial Cellulose Properties

BC structures are formed by extracellulary-excreted nanofibers produced by various species of bacteria, including *Acetobacter, Rhizobium, Agrobacterium and Sarcina* [17]. These nanofibers are about 100 nm in diameter and form a coherent 3D network. Macroscopically, the BC network is constructed as a pellicle that acquires the shape of the recipient where the bacteria is grown [18].

**Figure 1:**
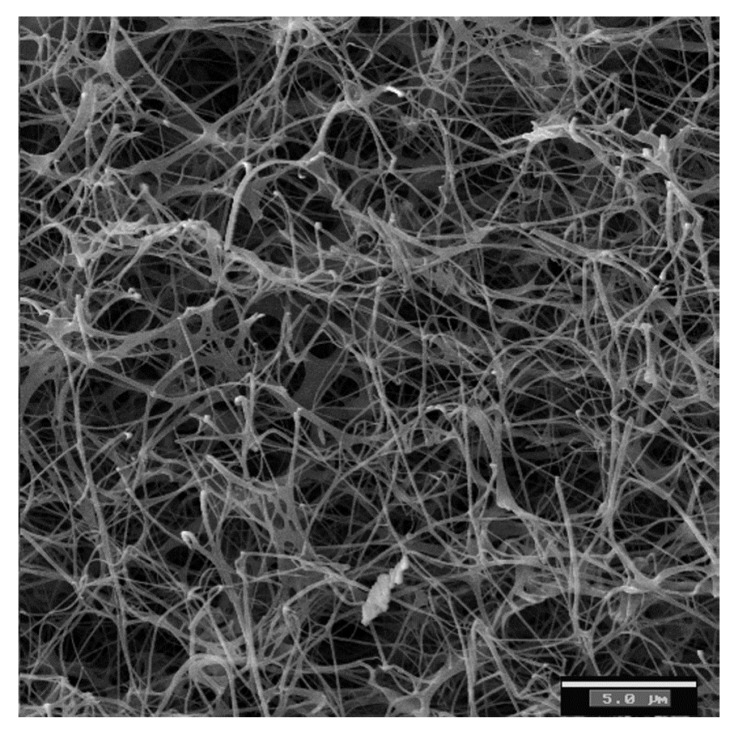
SEM micrograph of a bacterial cellulose sample showing a coherent 3-D network formed by cellulose fibers connected by physical joints [22].

The nanofibers of BC are ribbon-like structures of around 100 nm in diameter and around 100 µm in length. These ribbons are made up of bundles of cellulose microfibrils of 2–4 nm in diameter [2,3,19,20]. In its native state, BC is a water-swollen network of cellulose nanofiber (Figure 1). Figure 1 shows a SEM image of a native BC network. Grande *et al.* [10] have used image analysis to measure the morphological properties of dried BC networks. The average mesh size (distance between junction points) is 0.523 ± 0.273 µm, while the orientation (the average angle formed by the segments and the x-axis) of the nanofibers is 85.64 ± 0.56°.

The mechanical properties of these structures strongly depend on the properties of the network. Grande *et al.* [10] have reported mechanical properties of hot-pressed BC sheets. They have found an average Ultimate Tensile Strength (UTS) value of 241.42 ± 21.86 MPa, a maximum elongation of 8.21 ± 3.01% and a Young’s modulus of 6.86 ± 0.32 GPa. The elastic modulus of an individual BC nanofiber has been measured by means of Atomic Force Microscopy (AFM). Guhados *et al.* [21] have measured a value of 78 ± 17 GPa for Young's modulus of bacterial cellulose nanofibers with diameters ranging from 35 nm to 90 nm.

These remarkable mechanical properties are due to the uniform ultrafine-fiber network structure, the high planar orientation of the ribbon-like fibers when compressed into sheets and the good chemical stability of BC [23]. Applications include strong paper, headphone diaphragms, electro-acoustic transducers, high strength paper, components for precision optical devices, *etc.* [24,25].

BC networks can be used in its native state as a gel-like material, hot-pressed as a dry sheet or freeze-dried. They can also be used as part of a composite with another material. There are several reported processing routes for the production of BC composites. Some authors have tried to disintegrate the cellulose network structure in order to blend it as standard nanofiller [2,3,20,25].

Other routes consist in introducing a second phase during the development of the BC network. Touzel *et al.* [26] produced a double network gel of BC-pectin by adding pectin to the culture medium. Mormino *et al.* [27] have incorporated solid reinforcing particles in the forming BC gel by means of a rotating disc bioreactor.

**Figure 2:**
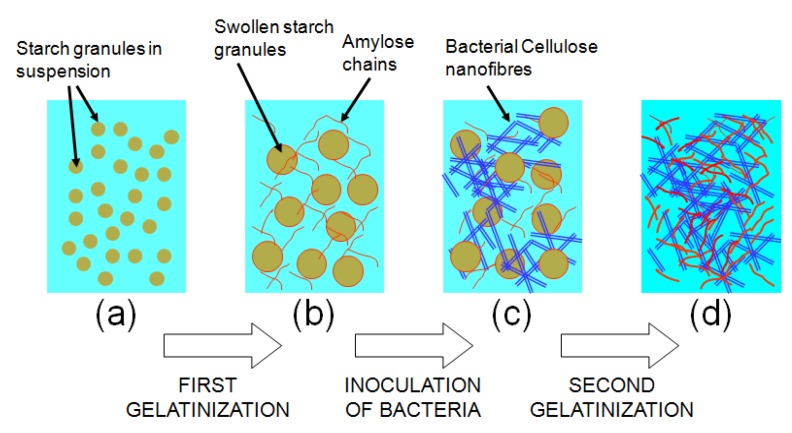
Scheme of the BC-starch Bottom-Up process: (**a**) Starch granules are in suspension in the culture medium; (**b**) After autoclaving, starch is partially gelatinized, amylose leaches and granules swell; (**c**) BC nanofibrils grow in presence of the partially gelatinized starch; (**d**) After hot-pressing, the nanocomposite shows interpenetrating networks of amylose and cellulose [23].

Grande *et al.* [23] have reported the introduction of starch in the BC network by modifying the BC culture media. This “bottom-up” route is depicted in Figure 2. Starch granules are added to the culture medium before bacteria are inoculated. Then, the starch-medium suspension is autoclaved at 121°. During this process, the starch phase undergoes a “first gelatinization”. The starch granules swell, leach amylose and lose their crystalline structure. When the starch-medium suspension cools down, bacteria are inoculated, and the nanofibers network is formed in the presence of a partially gelatinized starch structure. Finally, the BC-starch gels are hot-pressed, and the starch phase undergoes a “second gelatinization”.

## 3. Bacterial Cellulose as a Biomaterial

BC is used in the humid state for most biomedical applications. In this state, the BC network resembles a gel. BC gels are not easily rehydrated once they have lost water. Due to their irreversible nature, native BC gels have been named “never-dried BC” [28]. The mechanical properties of BC gels resemble the properties of soft tissues [29]. The reported stress at break is 2 MPa, with 99% water content.

Torres *et al.* [22] have measured the rheological properties of BC gels. They have found that both the storage modulus (G’) and the loss modulus (G’’) remain almost constant in the range of 10–100 °C. They have also found that the storage modulus of BC gels decreases as the applied stress increases, which is a typical behavior of physical gels due to the permanent alteration or even disruption of cross-links [30]. In addition, BC displays a “Reversible Stress Softening” type of behavior, in which the reduction of storage modulus is reversible. This “Reversible Stress Softening” has also been found in other biological gels, such as actin gels [31], and it is considered to be a protection mechanism.

According to Gatenholm and Klem [32], the use of BC gels for the fabrication of biomedical products has advantages over the use of other types of cellulose and polymers. These advantages include the fact that the 3D shape and the fiber network architecture can be controlled. Moreover, composite formation can be achieved in the culture medium, and the possibility of coating BC gels with foreign surfaces improves their biocompatibility.

Biocompatibility is the ability of a material to perform with an appropriate host response in a specific application [33]. Biocompatibility does not only refer to the quality of not having toxic effects on biological systems, but also to the need of having an appropriate host response to ensure satisfactory performance on a specific application. Petersen and Gatenholm [27] have pointed out that biocompatibility of BC for tissue engineering applications can be related to the fact that its structure shows similarities with extracellular matrix components, such as collagen. In fact, collagen and BC nanofibers have similar diameters (around 100 nm) and are extracellularly assembled from precursor molecules into polymer chains.

Mendes *et al.* [14] have assessed the tissue reaction in the presence of a BC membrane after subcutaneous implantation in mice. They performed analysis of histological sections of the BC membrane and the surrounding tissue at 7, 15, 30, 60 and 90 days post-surgery. They found no evidence of foreign body reaction throughout the study period. Polymorphonuclear cells and lymphocytes observed at 7, 15 and 30 days post-surgery suggest a mild inflammatory response. By contrast, at 60 and 90 days post-surgery, no inflammatory infiltrate was observed. Helenius *et al.* [15] implanted pieces of BC on rats. They also found no histologic signs of inflammation and no presence of giant cells. The formation of new blood vessels around and inside the implanted cellulose was observed. *In vivo* tests of tubular shaped BC membranes for the replacements of blood vessels have shown similar results. No macroscopic or microscopic signs of inflammation around the implants were found. Instead, it was found that fibroblasts had actually infiltrated BC [18,34].

Due to its biocompatibility, BC has been used as a scaffold for tissue engineering application [15,28]. Hutmacher [35] has identified several requirements that tissue engineering scaffolds should fulfill. Scaffolds should have interconnecting pores of appropriate scale to favor tissue integration and vascularization, appropriate surface chemistry to favor cellular attachment, differentiation and proliferation, adequate mechanical properties and should be made from materials with controlled biodegradability or bioresorbability so that tissue will eventually replace the scaffold.

Of all the requirements listed by Hutmacher, biodegradability seems to be the most difficult to meet for BC-based biomaterials. Cellulose has a high degree of crystallinity and a compact structure. Cellulase enzymes capable of performing cellulose hydrolysis are not present in animals, but are produced by fungi and bacteria. Li *et al.* [11] have reported the enhancement of the biodegradation of BC *in vitro* (in water, phosphate-buffered saline and simulated body fluid) through periodate oxidation. This chemical treatment kept the original network structure of BC intact and made it possible to prepare a BC-based scaffold that could degrade in water, phosphate buffered saline (PBS) and the simulated body fluid (SBF).

## 4. Improvement of Biocompatibility of BC Structures

**Figure 3:**
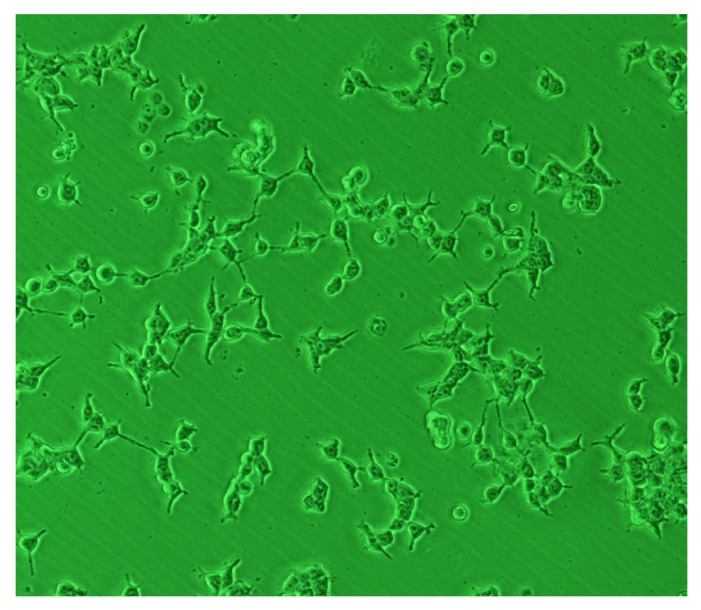
Optical micrographs of HEK cells for one day of culture in a composite of BC-Hidroxyapatite [12].

BC and BC-based composites have been used as scaffolds for cell seeding. Several studies have confirmed that different cells, such as human embryonic kidney cells (HEK) [12], bone forming osteoblasts (OB) [13], fibroblasts [13], chondrocytes [36], human smooth muscle cells (SMC) [28], *etc.*, can grow in the presence of BC. Figure 3 shows HEK cells growth on a BC based composite. However, cell adhesion cannot be achieved with native BC structures.

It seems that the surface of native BC does not promote cell adhesion. The interfacial characteristics of biomaterials play a key role in cell adhesion. The surfaces should promote the specific absorption of proteins and subsequent cellular interaction [37]. Several studies have been carried out with the aim of modifying BC surface to optimize the BC-cells interactions. These modifications change the physical properties of BC structures, such as wettability, porosity and surface chemistry. It has been shown that improvement of cell adhesion may be achieved by the immobilization of adhesion proteins onto the biomaterial surface [38]. However, the results show that these treatments have different results, according to the specific cells used.

Experimental evidence shows that surface modification of BC by nitrogen-containing plasma improves cell adhesion in the case of microvascular and neuroblast cells, but has no effect on fibroblast cells [39]. Pertile *et al.* [38] have used some of the small signaling peptides found in the proteins of the extra-cellular matrix, such as the integrin-ligand sequences isoleucine-lysine-valine-alanine-valine (IKVAV) fused to a carbohydrate-binding module (CBM3), in order to modify the surface of native BC with the aim of improving cell adhesion. These recombinant proteins were adsorbed to BC structures and were able to improve the adhesion of neuronal and mesenchymal cells, but had no effect on the other cell lines tested. Andrade *et al.* [40] have improved the adhesion of fibroblasts to BC. They managed to coat BC nanofibers with cellulose-binding module (CBM) fused to Arg-Gly-Asp (RGD). The results showed that fibroblasts exhibit improved ability to interact with BC sheets coated with CBM-RGD.

A different approach to improve BC biocompatibility consists in preparing BC composites with various biomaterials. Wang *et al.* [41] have reported the synthesis of a cellulose/gelatine composite via crosslinking by procyanidin. The results showed that the proliferation, infiltration and adhesion of fibroblasts are improved with regard to native BC. Indeed, gelatine, a polypeptide derived from an extracellular matrix, and a denatured form of collagen, exhibits many properties such as good biocompatibility, low immunogenicity, adhesiveness, promotion of cell adhesion and growth and low cost. Hence, it can be used as an alternative material to collagen in tissue engineering.

Collagen has also been used to improve BC biocompatibility. Collagen has been used in biomedical applications due to its biodegradability, low antigenicity and cell-binding properties [27]. By contrast, collagen has low mechanical properties, possible inter-infection and high price, thus limiting its use in biomedical fields [42]. Cai *et al.* [43] prepared BC-collagen composites by immersing a BC membrane in collagen solution. The incubated fibroblast cells performed well with regard to cell adhesion and proliferation. The BC-collagen composites prepared showed much better cytocompatibility than pure native BC.

BC-chitosan composites have been prepared in order to improve some of the properties of native BC structures. Kim *et al.* [44] have prepared BC-Chitosan composites by immersing wet BC pellicles in a chitosan solution followed by a freeze-drying process. The morphological analysis performed suggested that chitosan molecules can penetrate into BC forming a three-dimensional multilayer structure scaffold. The biocompatibility of such BC-Chitosan composites was evaluated by cell adhesion studies. Cell adhesion and proliferation was achieved. In general, the BC-chitosan composites showed much better biocompatibility with regard to pure BC.

Also, chitosan has been shown to improve some other important characteristics of BC. Ul-Islam *et al.* [45] prepared BC-Chitosan composites that showed improved water holding capacity (WHC) and water release rate (WRR). Cai *et al.* [46] reported the increase of thermal stability of BC-Chitosan composites with regard to native BC structures. They have found that the thermal degradation temperature increases from 263 °C to 366 °C, with the chitosan content increasing from 1.2% to 45%. By contrast, the tensile strength and Young’s modulus of BC-Chitosan composites tend to decrease with the increase in chitosan content, and the crystallinity tends to decrease from 82% (pure BC) to 61% (BC-Chitosan composites) [44].

Other biomaterials used to improve the biocompatibility of BC include starch [22,47], hydroxyapatite [12,16,48,49,50,51,52], poly(vinyl alcohol) [53,54,55], poly(methyl methacrylate) [56] and polyacrylamide [57].

Biocompatibility can also be improved by controlling the properties of BC-based structures. The porosity of BC networks can influence the response of cells. The density of the mesh of the BC networks could prevent cell migration into the biomaterial [27]. Porogens have been used to increase the porosity of BC networks. Backdahl *et al.* [58] used starch and paraffin wax as porogens. They were able to control the pore size and interconnectivity of pores in BC networks by controlling the size of the starch and paraffin wax particles used. Their tests showed that muscle cells were able to attach themselves inside the pores.

The control of porosity can also be used to modify the water holding capacity (WHC) and water release rate of BC structures (WRR). Both WHC and WRR determine the usefulness of BC as a dressing material [59]. The moisture content of a dressing material accelerates the wound healing process and protects wounds against contamination [60]. Dahman [61] claims that in the case of BC, its loose fibril arrangement, high surface area per unit mass and hydrophilic nature results in a very high WHC (100–200 times its dry weight). Rambo *et al.* [62] have reported the modification of porosity of BC networks by using a template with pins of different diameters (60–300 um).

## 5. BC Based Structures for Biomedical Applications

### 5.1. Bone Tissue Engineering and Bone Grafting

Bone is a composite material with an organic phase (collagen and noncollagenous proteins) and an inorganic mineral phase (calcium hydroxyapatite). Among the different materials used in bone tissue engineering and bone grafts, ceramics have been used extensively [63] due to their osteoconductivity. BC can be a good matrix for obtaining different types of calcium carbonate crystals with improved biocompatibility. Stoica-Guzun *et al.* [64] have used calcium chloride (CaCl_2_) and sodium carbonate (Na_2_CO_3_) as starting reactants to promote calcium carbonate deposition on bacterial cellulose membranes.

It has been proven that BC nanofibers can mimic collagen nanofibers for Ca-P minerals deposition via biomineralization. The resultant Ca-P minerals are platelet-like calcium-deficient hydroxyapatite (Hap), similar to the hydroxyapatite found in natural bone [65]. Other authors [48,51] have induced a negative charge on BC by the adsorption of polyvinylpyrrolidone (PVP) to initiate the nucleation of Hap via dynamic simulated body fluid (SBF) treatment. Shi *et al.* [16] introduced an alkaline treatment before the biomimetic mineralization process in order to improve the mineralization efficiency. Zhang *et al.* [66] have used a phosphorylation reaction to introduce phosphate groups to the hydroxyl groups of BC and promote the growth of calcium phosphate. Wan *et al.* [51] have also shown that phosphorylation effectively triggers Hap formation on BC. BC-Hap composites with a third phase can also be produced.

In order to improve the biocompatibility and osteoconductivity of BC in bone-related biomedical applications, Grande *et al.* [12] have produced BC-hydroxyapatite nanocomposites (Figure 3) by adding hydroxyapatite and carboxymethylcellulose (CMC) to the culture medium. As CMC can change the viscosity of media, it was used to retard the settlement of Hap particles in the BC culture medium.

### 5.2. Cardiovascular Clinical Applications

Synthetic blood vessels of other materials, such as Dacron® and expanded polytetrafluoroethylene are available at large diameters (6–10 mm). By contrast, small diameters vessels (<6mm) for coronary, carotid and femoral arteries are still under investigation [67]. Several studies have shown that BC can be molded into tubular form with diameter <6 mm [18,68,69]. Klemm *et al.* [18] designed a matrix in order to produce a BC tube (diameter 1 mm, length 5 mm, wall thickness 0.7 mm) with a tensile strength comparable to that of normal blood vessels (800 mN). Brown *et al.* [69] have prepared small tubes of BC-fibrin composites treated with glutaraldehyde in order to crosslink the polymers and allow a better match of the mechanical properties with those of native small-diameter blood vessels.

The biocompatibility of such BC-based blood vessels has been evaluated by *in vivo* tests. The tubes prepared by Klemm *et al.* [18] were used to replace part of the carotid artery (4–6 mm) of a rat. After four weeks, the BC/carotid artery complex was covered with connective tissue, showing that BC can be used as a replacement blood vessel.

BC-based blood vessels, known as BASYC®, have been tested in five mice. The BC implants were attached in an artificial defect of the carotid artery for one year. These BASYC® vessels were shown to be stable vascular conduits [34]. The tests showed that the BC-based blood vessels studied were biocompatible. Moreover, the morphological analysis carried out showed that structural modification in the contact region between the BC-based blood vessels and the surrounding media had occurred. The presence of fibroblasts in this region suggests that a process of integration without degradation (vitalization) has taken place.

The potential use of BC-based composites for the production of heart valve replacements is currently under study. Milton and Wan [55] prepared a BC-Poly(vinyl alcohol) composite that mimics the mechanical behavior of native heart valve leaflets. Their tests showed that the stress–strain properties for porcine aorta are matched by the one of the BC-Poly(vinyl alcohol) prepared in this study. Mohammadi [70] has also used BC-Poly(vinyl alcohol) composites for the production of heart valve leaflets. Mohammadi aimed at mimicking not only the non-linear mechanical properties displayed by porcine heart valves, but also their anisotropic behavior by applying a controlled strain to the samples, while undergoing low-temperature thermal cycling in order to induce oriented mechanical properties.

Hemocompatibility of surfaces that are in contact with blood should be further studied. It is well known that blood-contacting surfaces may activate coagulation and thrombus formation due to the interaction of such surfaces with blood proteins and platelets. Fink *et al.* [71] evaluated the hemocompatibility of BC-based vascular graft tubes and compared them with commercial grafts of poly(ethylene terephthalate) (PET) and expanded poly(tetrafluoroethylene)(ePTEE). They reported that BC-based grafts do not induce plasma coagulation, but induce the slowest coagulation cascade. Andrade *et al.* [72] have coated BC with the tripeptide Arg-Gly-Asp (RGD) to favor endothelialization and improve hemocompatibility of BC. They cultured human microvascular endothelial cells on RGD-coated BC. The results showed that the endothelial cells formed a confluent layer, inhibiting the adhesion of platelets.

### 5.3. Wound Dressings

BC gels have been used for wound dressings. According to Czaja *et al.* [9], the biocompatibility of BC-based wound dressings is related to its distinctive nanofibrillar structure, which serves as an optimal wound healing environment. This nanofibrillar structure eliminates pain symptoms (by isolating the nerve endings) and enhances the absorption of wound exudates [73]. BC accelerates the process of healing of the skin in comparison with conventional wound dressings, such as wet gauze and ointments. Studies have shown that BC-based coverings reduce wound pain, accelerate re-epithelization and reduce wound infection rates and scarring [9,68,74].

There are three commercially available BC-based coverings, namely Xcell®, Bioprocess® and Biofill®. Legeza *et al.* [75] produced a BC wound dressing for the treatment of third degree burns that is impregnated with superoxide dismutase (an antioxidant) or poviargol (an antibiotic).

### 5.4. Cartilage Replacement

Advanced degeneration of articular cartilage is often treated with the total replacement of joints by prostheses. Due to wear and osteolysis, the lifespan of these prostheses is limited. Some studies have shown that BC composites could be used as cartilage replacement material. Azuma *et al.* [76] concluded that BC-poly(dimethyl acrylamide) double network gel has mechanical properties similar to the mechanical properties of cartilage and that may meet the requirements of artificial cartilage. However, *in vivo* tests that could confirm the biocompatibility of BC-based cartilage replacements have not been reported yet. BC-poly(vinyl alcohol) composites evaluated using unconfined compression testing also have elastic modulus values similar to those reported for native articular cartilage [53].

### 5.5. Other Biomedical Applications

Other less-documented biomedical applications include the use of BC for the production of contact lenses [77], electroconductive composite hydrogels biosensors [78], membranes for topical delivery of lidocaine [79] and dietary supplements for combating diabetes [80].

## 6. Conclusions

Bacterial cellulose-based structures have been used for many biomedical applications, including bone tissue engineering, blood vessels and wound dressings, among others, since BC has proved to be a nontoxic biocompatible material. Some properties of BC, such as porosity, density, water holding capacity and its high strength make BC-based materials suitable for such biomedical applications. However, unmodified native BC is not always suitable for biomedical applications. For instance, cells tend to grow in the presence of native BC, but they do not attach themselves to an unmodified native BC substrate. In order to improve BC biocompatibility, several strategies have been proposed, including surface modification, porosity modification and preparation of BC composites. However, the physical stability and relatively low degradation rate of cellulose inside the human body could present challenges for BC to be able to be used in certain biomedical applications (such as scaffolds for tissue engineering). Further studies are needed in order to elucidate the way of promoting and controlling the biodegradation of cellulose. This could in fact facilitate the use of BC-based structures in other biomedical applications.
